# Mislabelling and high mercury content hampers the efforts of market-based seafood initiatives in Peru

**DOI:** 10.1038/s41598-020-77338-x

**Published:** 2020-11-23

**Authors:** Daniella Biffi, Andrea López-Mobilia, Shaleyla Kelez, Dean A. Williams, Matthew M. Chumchal, Molly Weinburgh

**Affiliations:** 1 Andrews Institute of Mathematics & Science Education, Texas Christian University, Fort Worth, 76129 USA; 2 ecOceánica, Lima, Peru; 3 Ruway, Lima, Peru; 4 Department of Biology, College of Science and Engineering, Fort Worth, 76129 USA

**Keywords:** Conservation biology, Environmental social sciences

## Abstract

Peru is experiencing a “gastronomic boom” that is increasing the demand for seafood. We investigated two implicit assumptions of two popular sustainable seafood consumer-based initiatives: (1) seafood is labelled correctly, and (2) the recommended species are healthy for consumers. We used DNA barcoding to determine the taxonomic identity of 449 seafood samples from markets and restaurants and analysed the concentration of total mercury (THg) in a sub-sample (271 samples) of these. We found that a third of seafood is mislabelled and that over a quarter of all samples had mercury levels above the upper limit recommended by the US EPA (300 ng/g ww). Additionally, 30% of samples were threatened and protected species. Mislabelling often occurred for economic reasons and the lack of unique common names. Mislabelled samples also had significantly higher mercury concentrations than correctly labelled samples. The “best choice” species compiled from two sustainable seafood guides had less mislabelling, and when identified correctly through DNA barcoding, had on average lower mercury than the other species. Nevertheless, some high mercury species are included in these lists. Mislabelling makes the efforts of seafood campaigns less effective as does the inclusion of threatened species and species high in mercury.

## Introduction

Recent attention on Peruvian cuisine has resulted in growth of the economic sector related to food, the so called “gastronomic boom”^1^. Specifically, demand for seafood has increased and there are now more than 12,000 *cevicherias* (seafood restaurants) in Lima alone^2^. At the same time, due to the health benefits of consuming fish and to increase job opportunities for artisanal fisherfolk, the Ministry of Production (the government agency that manages fisheries in Peru) started the “Let’s eat fish” campaign in 2012^3^. The campaign promotes the consumption of fish at least three times a week^4,5^. Meanwhile, the high demand for popular species like Fine Flounder (*Paralichthys adspersus*), Corvina Drum (*Cilus gilberti*), and Peruvian Grunt (*Anisotremus scapularis*), is believed to have negative effects on their populations since juveniles are being exploited to satisfy demand^6,7^.

In response to the increased demand for seafood, various market-based initiatives have been created. The initiatives, developed by non-profits, gastronomy associations, and government organizations, are intended to educate consumers and reduce the demand for unsustainable seafood. Two popular sustainable seafood guides in Peru are *El tamaño sí importa* (Size does matter, hereafter SDM) and *bóVEDA*. These include a list of species to avoid but generally define sustainable consumption as the consumption of species above their minimum landing size^8,9^.

Therefore, these initiatives are based on two implicit assumptions. First, that there is no seafood mislabelling. Mislabelling is the practice of misrepresenting the identity of fish (or other seafood) to consumers, it occurs mainly for economic reasons and appears to be widespread^10^. Mislabelling can result in consumers inadvertently eating “at risk” species. Weak regulation and monitoring at landing sites^11^, a lack of unique vernacular names^12^, and the high demand for certain species creates an environment for mislabelling to occur in Peru. Second, seafood campaigns assume that the recommended seafood represents a healthy choice for consumers. Mercury (Hg), is a globally distributed toxic metal, that biomagnifies up the food web reaching high concentrations in fish^13^. The main source of MeHg in humans is through the consumption of seafood^14^. A lack of contaminant monitoring in marine species has resulted in limited information about the safety of species featured in sustainable seafood campaigns^15,16^. Assumptions that seafood is correctly labelled and low in contaminant concentrations need to be verified or they can render the efforts sought by campaigns, such as changes in buying behaviour, ineffective while putting consumers at risk. Our study explored these two implicit assumptions of sustainable seafood campaigns. We determined the presence and extent of seafood mislabelling and Hg concentration in 449 and 271 samples of fish respectively, from markets and restaurants in Lima and Tumbes, Peru.

## Results

### Mislabelling

Of the 449 DNA barcodes generated, four samples were identified to order, four to family, 13 to genus, and 428 to species (Supplementary Table S1). We found that 32.7% (147 out of 449 samples) were mislabelled (Supplementary Table S1 and S2 for all the common names used for each species). We were able to buy 25 of the 36 “best choice” species recommended by the SDM and bóVEDA campaigns (n = 157 samples) (Supplementary Table S3). Barcoding revealed that 14.7% of these samples were mislabelled (n = 23). Of these 23 mislabelled samples, over half (n = 12) were other “best choice” species. Mislabelling was lower for “best choice” species than all other species (42.5%, 124 of 292 samples) (Fisher’s exact test, *P* = 2.7 × 10^–9^).

#### Mislabelling by venue

**Figure 1:**
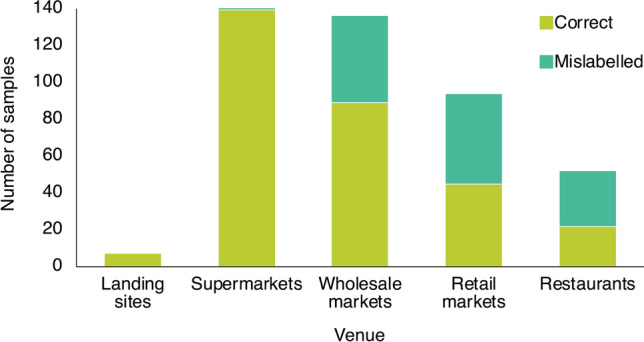
Number of mislabelled samples by venue (N = 449; Landing sites = 7; Supermarkets = 160; Wholesale markets = 136; Retail markets = 94; Restaurants = 52).

**Figure 2:**
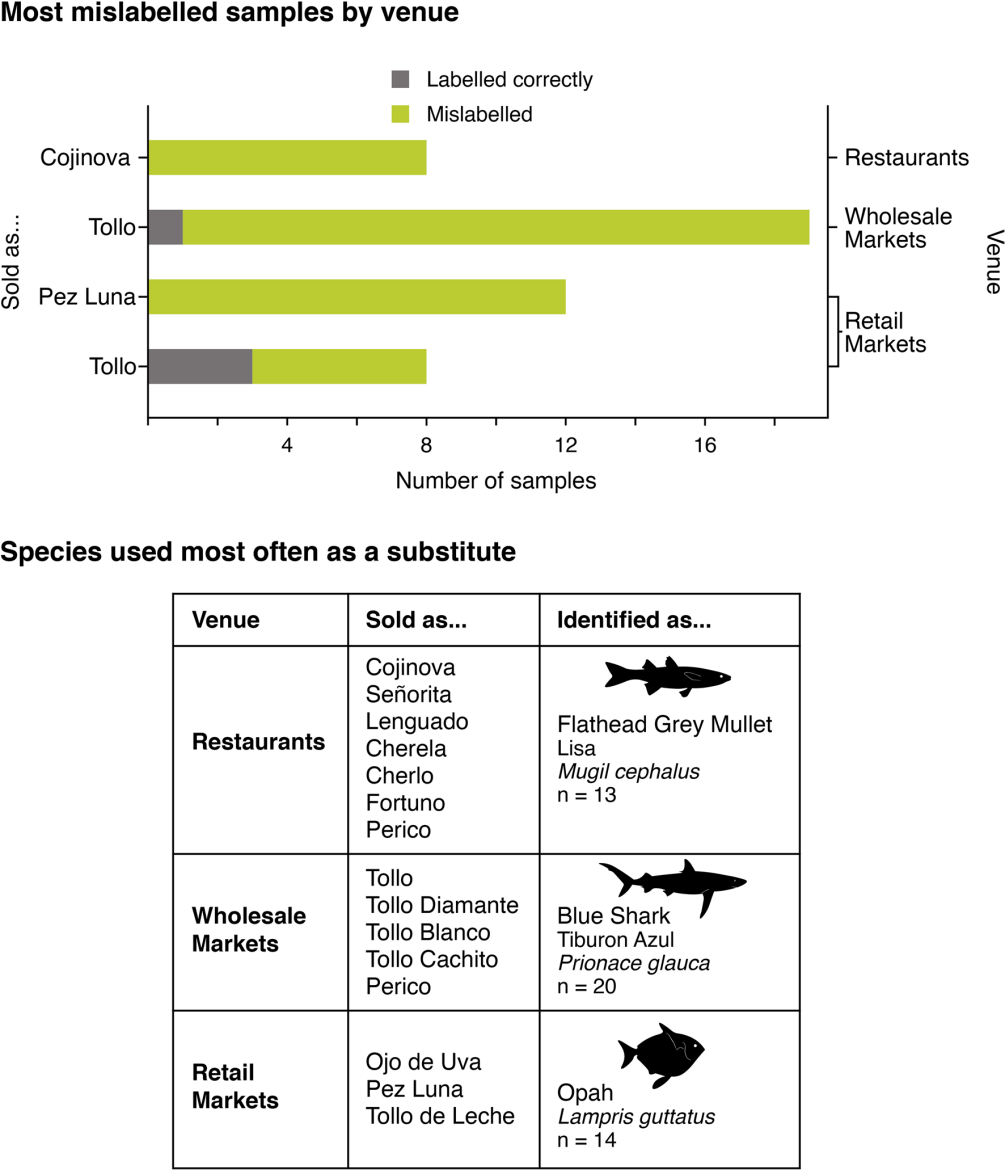
Most mislabelled species at restaurants, wholesale markets, and retail markets. For a complete list of scientific and common names in English and Spanish see Supplementary Table S2.

Mislabelling was higher than expected by chance in restaurants and retail markets, while mislabelling occurred at expected levels in wholesale markets, and less than expected in supermarkets (*Chi*-square = 58.4, df = 3, *P* < 0.001) (Fig. 1). The species most frequently mislabelled at restaurants was Cojinova (common name for *Schedophilus haedrichi* and *Seriolella violacea*). The species used most often as substitutes in restaurants were Flathead Grey Mullet (*Mugil cephalus*) and Tilapia (*Oreochromis* spp.), and they were sold as seven different species. The species most frequently mislabelled in retail markets were Pez Luna (*Mola mola*) and Tollo, and the species used as a substitute most often was Opah (*Lampris guttatus*). At wholesale markets, the most mislabelled species was Tollo and the species used as a substitute most often was Blue Shark (*Prionace glauca*) (Fig. 2).

#### Mislabelling by price

**Figure 3:**
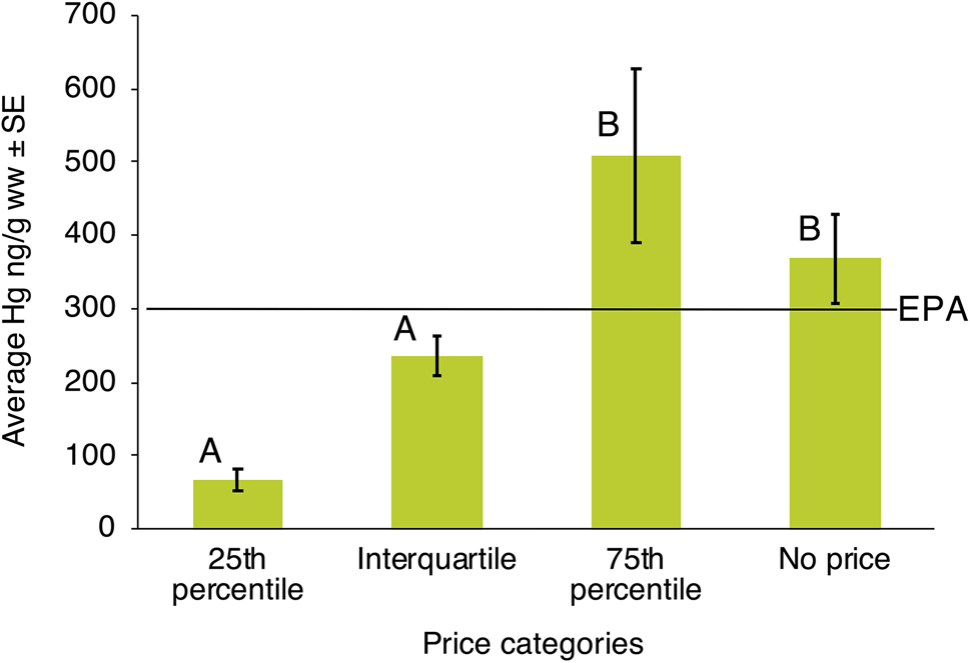
Average ± SE mercury content by price (Peruvian Nuevos Soles [PEN] per kilogram). Samples were grouped into four categories based on price: Below the 25th percentile (1–4.3 PEN); Interquartile (4.6–12.3 PEN); Above the 75th percentile (13.6–42.5 PEN); and No price available. Different letters above bars indicate which averages are significantly different using Tukey’s post-hoc tests.

The advertised fish was replaced by a lower value fish 67 times, a higher value fish 22 times, and an equal value fish 10 times (*Chi*-square goodness of fit test = 54.73, df = 2, *P* < 0.001). The advertised fish was replaced by a lower value fish significantly more than expected by chance (n = 67) (*Chi*-square goodness of fit test = 35.0, df = 1, *P* < 0.001) (Fig. 3). The average value of mislabelled samples was also lower (8.64 ± 0.52 PEN) than the advertised price (15.37 ± 1.08 PEN) (paired samples t-test; t_98_ = 5.51, *P* < 0.001).

#### Mislabelling—protected species

Nearly one third of all the samples collected were identified as either near threatened (20.5%, n = 92), vulnerable (3.8%, n = 17), or endangered (6.0%, n = 27)^17^. Twenty-nine (31.5%) of the near threatened samples, 13 (76.5%) of the vulnerable samples, and three (11.1%) of the endangered samples were mislabelled. In eight cases these species were mislabelled as a less threatened species, however, in seven cases the species was sold as an even more threatened species than the substitute. The remaining 30 mislabelled samples were all sold as Tollo, Tollo Bebe, or Tollo de Leche which includes species of less conservation concern but also some species in threatened categories.

From the 92 samples identified as near threatened, most samples (n = 52) were Blue Sharks and half of these (n = 26) were mislabelled. Yellowfin Tuna (*Thunnus albacares*) comprised the second most common near threatened species with 36 samples, only two of which were mislabelled. Samples identified as vulnerable belong to five different shark species and two bony fish. All seven samples of the Smooth Hammerhead Shark (*Sphyrna zygaena*), a species classified as vulnerable^17^, were mislabelled as other shark species. All samples identified as endangered were Shortfin Mako (*Isurus oxyrinchus*), 24 of which were labelled correctly and seven were mislabelled and substituted with other sharks and billfish.

Two species that are currently protected under Peruvian laws were mislabelled most of the time. Eight samples sold as Aguja, Pez Aguja, or Pez Aguja Blanca (Needle, Needlefish, or White Needlefish) were identified as the protected Black Marlin (*Istiompax indica*, n = 6) and Indo-Pacific Sailfish (*Istiophorus platypterus*, n = 2)^18^. Another sample mislabelled as Needlefish was identified as Blue Marlin (*Makaira nigricans*). Blue Marlin is considered vulnerable by the IUCN^17^ and is not included in the Annotated Marine Fish Catalog nor in the decree that protects other billfishes. Only one sample of Striped Marlin (*Kajikia audax*) that was obtained at an artisanal landing site was correctly labelled as Marlin. On the other hand, two samples labelled as Marlin were identified as Wahoo (*Acanthocybium solandri*). One sample labelled correctly as Muchame (commercial name used in Peru for fresh or salt-dried small cetacean meat) was identified as the protected Short-beaked Common Dolphin (*Delphinus delphis*)^19^.

### Mercury

**Figure 4:**
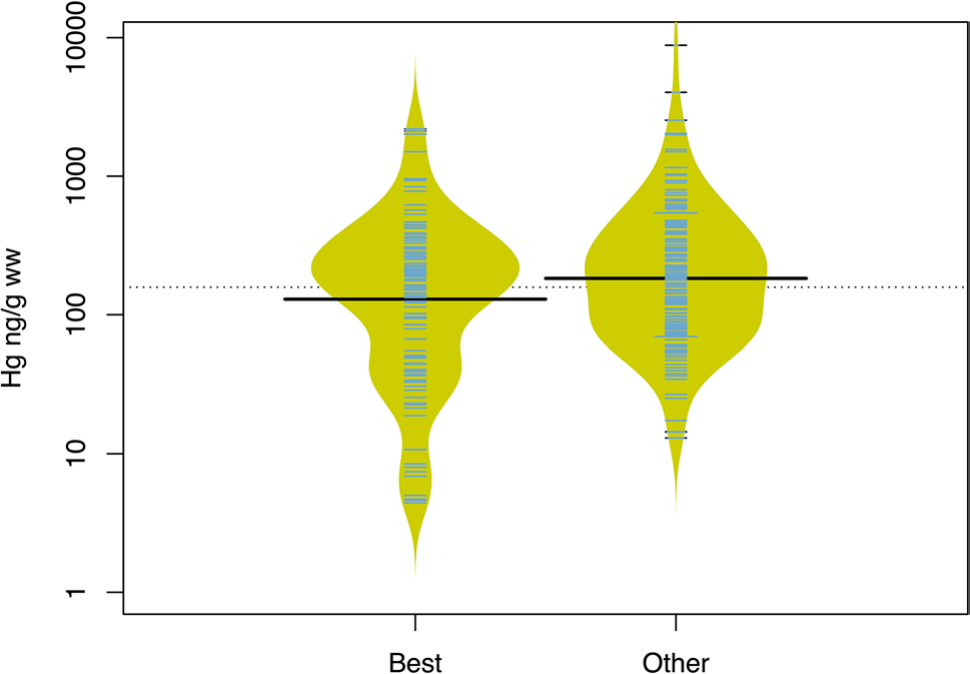
Beanplots of the mercury concentrations of samples identified as “best choice” and as other species. “Other” refers to samples classified as “species to avoid” and species not included in the guides. Each “bean” shows the distribution of mercury concentrations in Hg ng/g ww. The y-axis is log transformed. The black lines in each bean indicate the average mercury concentration for each group. Each blue horizontal line represents a sample. The dotted line indicates the overall average mercury concentration for all samples (331 Hg ng/g ww, n = 271).

A subset of 271 samples representing a total of 45 species, one genus, and two orders, were analysed for total mercury content (hereafter mercury; for full explanation go to Mercury methods). Mercury concentrations averaged 331 ± 41.3 ng/g wet weight (ww; range 4.42–8813 ng/g ww) and 28.0% (n = 76) of samples had mercury levels above the United States Environmental Protection Agency (US EPA) screening value of 300 ng/g ww^20^. There was a trend for the average mercury level to be lower in samples bought as best choice (281.6 ± 43.7 ng/g ww, n = 91) than in all other samples (356.0 ± 58.1 ng/g ww, n = 180) (t_150_ = 1.90, *P* = 0.06). Best choice samples as identified through barcoding did have significantly lower mercury than all other samples (267.0 ± 35.2 ng/g ww, n = 115 versus 378.2 ± 66.7 ng/g ww, n = 156) (t_216_ = 2.25, *P* = 0.03) (Fig. 4).

#### Mercury by species

Samples from 20 different species had mercury concentrations above the US EPA screening value. Predatory fish such as billfish, sharks, Wahoo, Orangemouth Weakfish (*Cynoscion xanthulus*), Opah, and *Centropomus* had the highest average levels of mercury and they were all above the US EPA recommended level (Table 1).

**Table 1:** Summary of the samples analysed for mercury content. Samples marked by (*) represent species with average (or single sample) mercury concentrations above the US EPA guideline of 300 ng/g ww.

Species	N	Hg mean	Std. error	Min	Max	N samples > 300 ng/g ww Hg
Istiompax indica*	4	4094.77	1664.30	1504.14	8813.47	4
Makaira nigricans*	1	762.63				1
Xiphias gladius*	13	391.02	69.52	85.57	1012.58	8
*Acanthistius pictus*	1	263.48				
*Anisotremus scapularis*	1	274.27				
*Brotula clarkae*	10	275.56	41.75	96.53	466.96	4
*Cilus gilberti*	11	112.26	26.59	25.15	297.41	
*Coryphaena hippurus*	4	93.61	35.60	14.35	164.26	
*Hyporthodus acanthistius*	2	99.65	15.90	88.41	110.89	
*Hyporthodus niphobles*	3	263.30	102.67	60.86	309.36	2
*Paralichthys adspersus*	1	118.59				
*Paralichthys woolmani*	10	272.50	50.58	14.42	464.29	5
*Seriolella violacea*	6	60.44	13.93	23.18	101.90	
*Thunnus albacares*	24	201.75	18.92	66.05	443.94	1
Callorhinchus callorynchus*	1	350.76				1
*Myliobatis chilensis*	3	220.62	104.60	12.99	346.62	2
Alopias pelagicus*	1	1557.79				1
Alopias superciliosus*	1	895.65				1
Alopias vulpinus*	2	595.93	789.34	37.78	1154.07	1
Isurus oxyrinchus*	22	607.90	139.96	85.15	2190.73	12
Mustelus lunulatus*	1	587.22				1
Mustelus sp.	10	88.86	5.87	69.45	121.38	
*Prionace glauca*	40	296.14	40.07	21.40	1501.84	13
Sphyrna zygaena*	5	786.01	452.60	148.90	2534.00	2
Acanthocybium solandri*	4	587.60	116.45	426.52	928.86	4
*Aplodactylus punctatus*	1	17.37				
*Caulolatilus affinis*	1	49.76				
*Cyclopsetta querna*	2	97.34	18.80	84.04	110.63	
*Cynoscion praedatorius*	3	108.64	43.75	41.77	190.94	
Cynoscion xanthulus*	9	333.04	117.74	53.73	1035.87	3
*Delphinus delphis*	1	55.09				
*Diplectrum conceptione*	3	48.81	13.11	26.81	72.17	
*Dosidicus gigas*	3	41.97	5.11	34.48	51.73	
*Hemanthias signifer*	3	84.65	25.85	41.69	131.03	
Lampris guttatus*	15	481.67	121.23	70.26	1994.83	8
*Lobotes pacificus*	1	97.79				
*Merluccius gayi*	3	114.92	42.64	36.52	183.17	
*Mugil cephalus*	10	9.31	2.70	4.42	32.85	
*Odontesthes regia*	1	18.85				
*Palabrax humeralis*	2	64.34	11.05	56.53	72.16	
Perciformes: Centropomus sp.*	2	477.62	186.98	345.41	609.83	2
*Prionotus stephanophrys*	6	55.57	19.17	28.77	150.78	
*Sarda chiliensis*	15	96.62	20.44	22.56	297.58	
*Schedophilus haedrichi*	4	108.24	15.28	79.18	136.00	
Sciaenidae: Cynoscion parvipinnis	2	143.17	119.29	58.82	227.52	
*Sebastes oculatus*	1	177.83				
*Sphoeroides annulatus*	1	158.79				
*Trachinotus paitensis*	1	40.18				

**Figure 5:**
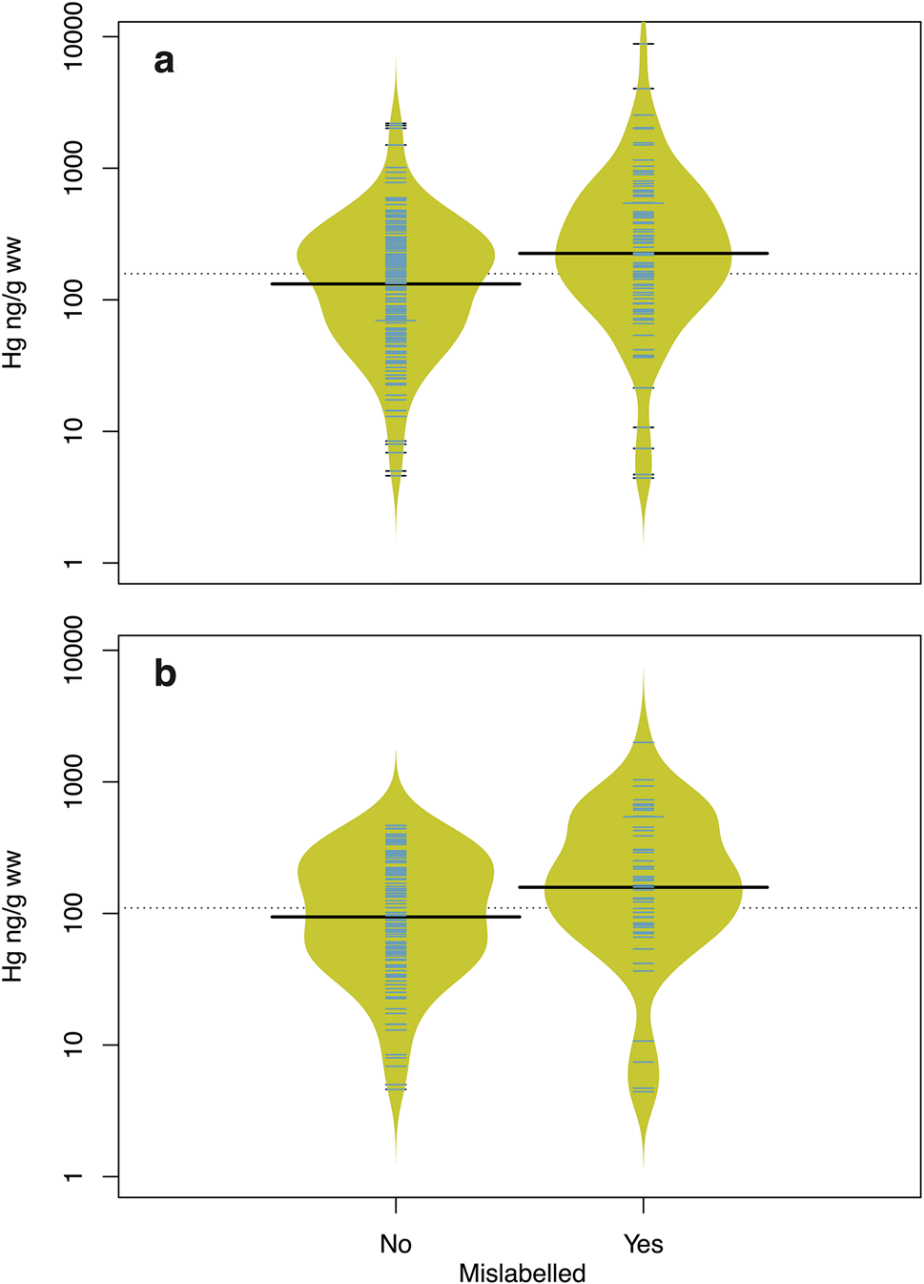
Beanplots comparing mercury concentration of samples labelled correctly (No) and mislabelled (Yes). (**a**) Total mercury concentration for all samples (331 Hg ng/g ww, n = 271). (**b**) Total mercury concentrations for all samples excluding sharks and billfishes (194 Hg ng/g ww, n = 171). Each “bean” shows the distribution of mercury concentrations in Hg ng/g ww. The y-axis is log transformed. The black lines in each bean indicate the average mercury concentration for each group. Each blue horizontal line represents a sample. The dotted line indicates the overall average mercury concentration for the samples included in the analysis.

Average mercury levels were higher for mislabelled samples (523.9 ± 111 ng/g ww, n = 91) than samples that were correctly labelled (233.5 ± 23.6 ng/g ww, n = 180) (t = 3.26, df = 155, *P* = 0.001) and there was a higher proportion of mislabelled samples (n = 39) that were above the US EPA screening level than correctly labelled samples (n = 37) (Fisher’s exact test, *P* = 0.0001) (Fig. 5a). After removing all sharks and billfish from the data set, average mercury levels were still higher for mislabelled samples (299.8 ± 48.3 ng/g ww, n = 52) than correctly labelled samples (148.1 ± 11.5 ng/g ww, n = 119) and there was still a higher proportion of mislabelled samples (n = 17) that were above the US EPA screening level than correctly labelled samples (n = 15) (Fisher’s exact test, *P* = 0.002) (Fig. 5b). These higher mercury levels were in large part due to the mislabelling of Opah, Orangemouth Weakfish and Wahoo in market places. In restaurants, substitutes were often species lower in mercury such as Flathead Grey Mullet and Tilapia.

Average mercury levels were similar between mislabelled (222.8 ± 87.4 ng/g ww, n = 12) and correctly labelled samples (290.6 ± 48.7 ng/g ww, n = 79), for samples bought as “best choice” species compiled from seafood guides (t_12_ = -1.13, *P* = 0.30) and the proportion of mislabelled samples that were above the US EPA screening level (n = 3) was the same as correctly labelled samples (n = 17) (Fisher’s exact test, *P* = 0.30).

#### Mercury by price

Hg levels were significantly different between price categories (ANOVA, *F*_3,267_ = 9.61, *P* < 0.001). Hg levels were significantly lower in the 25th percentile and interquartile price categories than in the 75th percentile and the no price categories (Tukey post-hoc tests). The 75th percentile and the no price categories had average values above the US EPA guidelines (Fig. 3).

## Discussion

We found that a third of seafood is mislabelled and that threatened and protected species, and species high in mercury are commonly sold in restaurants and markets. The “best choice” species that we compiled from the bóVEDA and SDM guides had less mislabelling than the other species we sampled and when mislabelled it was often replaced by another “best choice” species. Our results however, also suggest that government regulations and recommendations of seafood campaigns need to be revised to consider species in IUCN threatened categories and species high in mercury.

### Mislabelling

We found that 33% of the samples collected were mislabelled, similar to two other recent studies in Peru (27%^21^ and 43%^22^). Mislabelling in Peru is likely due to a variety of reasons including economic factors, the lack of an official name for each species, the existence of different local names for a single species, the inability to identify the species correctly based on morphology, and to conceal the trade of illegal species or species with some degree of protection.

Similar to other studies we found that mislabelled samples were usually replaced with lower value species^10^. Surprisingly, some of the most commercially desirable species were mislabelled at relatively low levels. For instance, of the 24 samples sold as Lenguado (*P. adspersus*), one of the most desired species in Peru, only four (16.7%) were mislabelled. In contrast, other studies have found that high demand species are substituted with species of lower commercial value at higher rates^10^.

It has been suggested that chefs in Peru (mainly those from high-end restaurants) could be ambassadors for sustainable seafood consumption^7^. And chefs have in fact helped in the promotion of more sustainable options (e.g., Peruvian anchoveta^23^). The restaurants sampled in this study were featured as “responsible restaurants” in the bóVEDA app or are in the same price range and are usually promoted as engaging in sustainable practices^9,24,25^. Nevertheless, 72.2% of the restaurants (13 out of 18 restaurants) used fish substitutes and more than half of the samples obtained at restaurants were mislabelled, usually with a cheaper species. This result is consistent with previous studies which found 38%^21^, and 61%^22^ of restaurant samples to be mislabelled.

Some mislabelling was likely due to the fact that many species do not have a single common name. Common names are currently not regulated in Peru or in fact in most places around the world^10^. For example, most shark species in Peru do not have a unique common name and are instead referred to interchangeably as “Tollo” or “Tiburón’. Nevertheless, Tiburón (e.g., *P. glauca*, *I. oxyrinchus*, *S. zygaena*, *Alopias* spp.) has a negative connotation due to their ammonia odour, while Tollos (e.g., *Mustelus* spp.) are considered more desirable by consumers^26^. In this study, Blue Sharks had high rates of mislabelling (49.1%) which may be explained by the negative perception towards this species because of its characteristic flaccid meat (which gives it the common name “Tiburón Aguado” [“soggy shark”])^27^. The use of misleading common names for sharks is not unique to Peru. In Italy, Blue Sharks are purposely sold as ‘Palombo’, a name that refers to the more valuable Smoothhounds^28^. In Brazil, the term ‘Cação’ is used instead of “Tubarão” (shark) since the latter term has a negative connotation^29,30^.

Moreover, similar to previous research^11,29^, not all cases of mislabelling identified in this study can be considered purposeful but are instead the result of incorrect assignment of common names. For example, all 17 samples purchased as Ocean Sunfish, from four different venues, were in fact Opah. Similarly, all five samples obtained as Doncella from four different venues were in fact Damsel Bass (Princesa, *Hemanthias signifer*).

Thirty percent of the samples we collected belonged to species that are listed as endangered, vulnerable, and near threatened by the IUCN and do not receive any special protection in Peru. Therefore, these species are sold openly, and maybe because of this, many of these were usually correctly labelled (i.e., Shortfin Mako, 3 out of 27 samples were mislabelled). A previous study found that the Smooth Hammerhead was being captured during closed season^21,31^. In this study, Smooth Hammerheads were all mislabelled despite being commercialized when the seasonal harvest ban was not in effect. It would be worrisome if fisherfolk, and ultimately fishmongers, are mislabelling Smooth Hammerheads year-round due to the special regulations established by the government (i.e., catch quota and seasonal harvest ban^31^). Recent research found that such restrictions, although needed, were deemed unfeasible for fisherfolk due to the non-selectivity of the gear used (e.g., gillnets^32^). We also found evidence that species protected under Peruvian laws are still being captured and commercialized (e.g., billfishes, dolphins). Shark species commercialized as Tollo or Tiburón had a high rate of mislabelling (38.5%), and 90% of the substituted species were other species of sharks. Of the nine species of sharks identified, six are classified as endangered or vulnerable^17^. Finally, the “best choice” list includes three species that are currently classified by the IUCN as near threatened and one species classified as endangered. Most of these species are also high in mercury, and so from the standpoint of consumer’s health should be listed as avoid.

### Mercury

Mercury concentration in fish varied greatly between samples but was generally highest in predatory fish as found in previous studies^33^. In this study, we found that mislabelling can potentially expose consumers to species with high mercury levels. This appears mainly to be a problem for species in market places since substitutions in restaurants were often cheaper species such as Flathead Grey Mullet that are low in mercury. Correct identification of “best choice” species using DNA barcoding also revealed that they are on average lower in mercury than other species. Most of these “best choice” species are fish at lower trophic levels which are expected to have lower levels of THg. The percentage of MeHg in THg has also been found to be lower in fish at lower trophic levels such as forage fish^34–36^. Nevertheless, the “best choice” list includes eight species of predatory fish that are high in mercury (Supplementary Table S3). Seafood campaigns often focus on minimum sizes in their recommendations to avoid the exploitation of juveniles which can negatively impact populations. Targeting the size or weight of the fish is generally not feasible since consumers usually do not encounter the ideal environment for measuring seafood at markets or restaurants. Often, fish are in display cases, already cut into pieces, or cooked. These size recommendations also do not consider that THg and the percentage of MeHg in muscle often increase with the size and age of the fish^35^. A better strategy would be to simply remove predatory species that are expected to be high in mercury from these lists.

In Peru, mercury levels are not monitored or regulated in seafood. There is an official health advisory that restricts the consumption of Zamurito (*Calophysus macropterus*), a freshwater catfish species. This species has levels of mercury between 520 and 890 ng/g ww^16^. In this study, billfish, sharks, Wahoo, Orangemouth Weakfish, Opah, and *Centropomus* in many cases had higher concentrations of mercury than Zamurito. Some samples had concentrations nearly 10 times higher than Zamurito; however, there is no recommendation from the government to regulate the consumption of species in these groups. Individuals, especially children and women of childbearing age may be at risk if they consume some of these species on a regular basis. For instance, using the US EPA reference dose (RfD, the amount of mercury that a person can safely consume each day) for mercury of 0.1 μg/kg/day^37,38^ the safe dose for a 60 kg woman would be 0.1 × 60 kg = 6 µg of MeHg per day or 42 µg a week. If this woman consumes 113 gr (raw fish recommended serving size for an adult^39^) of shark meat once a week and we use the mercury values obtained in this study for sharks, she would be consuming between 2.4–286.3 µg of mercury. These values fall below the recommended limits to almost seven times the recommended amount of mercury for a week. Some studies have suggested that the RfD for women of childbearing age should be below the current recommendations (e.g., 0.06 μg/kg/day^37^) which would mean women would exceed the recommended RfD by 1.8X to 11X.

### Conclusions

Seafood recommendations aimed at consumers in Peru should be revised to consider threatened species, content of mercury, price, and availability. Ideally, there also needs to be better government regulation of the seafood industry by establishing a list of unique common names, protection of endangered and threatened species, and creating health advisories for species high in mercury. While the “Let’s eat fish” governmental campaign emphasizes the consumption of forage species three times per week like Peruvian Anchovy, Jack Mackerel, Horse Mackerel, Chilean Silverside, and Flathead Grey Mullet as the most nutritious species available^40^, their messages exclude information about what species to consume in moderation or avoid altogether. A shift in fish consumption to forage species, not only would be beneficial for the marine ecosystem, but would also be beneficial to consumers since these species are cheaper, represent a healthier alternative, and are less likely to be mislabelled. However, this will require an integrated approach to the problem that encompasses actions along the entire supply chain, education campaigns aimed at consumers, fisherfolk, restaurateurs, and other groups of interest, development of adaptive strategies for fisherfolk through participatory processes, and consideration of global issues like the high demand of shark fins.

## Methods

### Sample collection

Tissue samples were collected between May and June 2017, in the Peruvian coastal regions of Lima and Tumbes. At the time of collection, there were no studies on seafood mislabelling in the country, so we collected samples from all the species present at markets and restaurants. We collected a total of 449 samples, representing a minimum of 64 different species. One-hundred and sixty samples (35.6%) were collected from 18 supermarkets, 136 samples from two wholesale markets, 94 samples (20.9%) from four retail markets, 52 samples (11.6%) from 18 restaurants, and seven samples (1.6%) from two artisanal fisheries landing sites (Supplementary Table S1). In Peru, retail markets (*mercados de abastos*) are small, local grocery stores; supermarkets are bigger, usually belong to a retail chain, and offer a larger variety of products; wholesalers are specialized venues that provide fresh seafood mainly to retail markets and restaurant intermediaries, and to a lesser extent to households^41^.

In most cases, samples were purchased as a whole fillet or fillet that had been chopped into small pieces. Four samples were taken from whole individuals that had not yet been processed (three fin samples and one tongue sample). Sampling efforts in restaurants were focused on those included as ‘responsible restaurants’ in the bóVEDA app^9^ and restaurants surveyed in a previous study^42^. Other restaurants not included in these lists, but with a similar price range, were also surveyed.

All samples were placed in a vial with 1 ml 8 M urea preservative buffer (10 mM Tris pH 7.5, 125 mM of NaCl, 10 mM EDTA pH 8.0, 1% SDS and 8 M urea)^43^. Preserved samples could not be used for mercury analysis since the preservative lowered mercury concentrations due to the presence of EDTA^44^. Therefore, a subset of 271 muscle samples were collected and frozen at − 20 °C without preservative.

### DNA methods

For DNA extraction, a 5 mm^2^ piece of tissue was separated from each sample and was placed in 400 μl of lysis buffer (75 mM NaCl, 25 mM EDTA, 1% SDS) with 15 μl of Proteinase K (20 mg/ml) and incubated overnight at 60 °C. The next day, 0.5 volumes of 7.5 M ammonium acetate was added and proteins were pelleted by centrifugation for 10 min. The supernatant was transferred to a new tube and 0.7 volumes of isopropanol was added to precipitate the DNA which was pelleted by centrifugation for 15 min. The pellet was then washed with 70% ethanol, vacuum dried, then resuspended in 200 µL 10 mM Tris–HCl pH 8.5.

For the cooked samples collected from restaurants, DNA was extracted using DNeasy mericon Food Kit (Qiagen, Valencia, CA, USA). Negative DNA extraction controls were included in each extraction batch to ensure non-contamination of reagents. To minimize contamination, DNA extractions were conducted in an extraction dedicated AirClean Systems 600 PCR workstation.

After the DNA extraction, a portion of the cytochrome oxidase I (COI) gene was amplified in all samples using the primer cocktail COI-3 for fish^45^. PCR reactions (10 μl) contained 5 μl of AccuStart II PCR SuperMix (Quanta BioSciences, Gaitthersburg, MD, USA), 0.5 μl of primers, 0.2 μl of BSA, 3.3 μl of water, and 1 μl of extracted DNA. Samples were run on an ABI 2720 thermal cycler using the following PCR profile: 94 °C for 10 min, then 30 cycles of 94 °C for 30 s, 50 °C for 15 s, 72 °C for 1 min., and then a final extension at 72 °C for 5 min. PCR products (4 µl) were checked on 1% agarose gels for amplification, and then the remaining product was enzymatically cleaned with *ExoI* and *rSAP* using manufacture’s protocols (New England Biolabs). These products were then sequenced bi-directionally with the primers M13-27 and M13-21 using BigDye Terminator Cycle Sequencing kit v.3.1 (Applied Biosystems) and electrophoresed on an ABI 3130XL Genetic Analyzer (Applied Biosystems). The sequences were first trimmed to remove primer sequences and base calls that had quality values below 25 using Sequencher (v. 5.0). The trimmed sequences were put into contigs using Sequencher and all had base calls with quality values between 20 and 40. All chromatograms were also checked by eye to determine if there were any ambiguities in base calls between the forward and reverse sequences. Sequences were all translated using MEGA 10^46^ to check for stop codons which would indicate the presence of pseudogenes.

### Determination of mislabelling

Sequences ranged from 80 to 616 base pairs (average length 546.3 bp, SE ± 3.5) (Supplementary Table S1). We submitted the sequences to Barcode of Life Data System (BOLD; https://www.boldsystems.org) to identify species. When a sample could not be identified in BOLD (n = 11 samples) we submitted the sequence to the Basic Local Alignment Search Tool (BLAST; https://www.blast.ncbi.nlm.nih.gov/Bast.cgi). A sample was identified to species level with ≥ 98% match, to genus level with ≥ 94.9% match, to family level with ≥ 91% match, and order level with ≥ 85.9% match^29^. Scientific names and English common names of fish were verified using FishBase (fishbase.org)^47^. The conservation status of species was determined from the IUCN Red List (Version 2019-3^17^).

To determine if there was mislabelling, we used the “Annotated Marine Fish Catalog from Peru” (Catalogo Comentado de los Peces Marinos del Peru) from the Peruvian Marine Institute (IMARPE)^12^. Although there are other field guides that contain additional common names for sharks^48–50^, the Catalog is the most complete list of marine species that includes Spanish common names used in Peru.

We compared the DNA barcode identification to the common name(s) for each species in the Catalog. Usually the Catalog featured more than one common name for each species. We considered a sample to be mislabelled if the name used at the venue did not match any of the common names used in the Catalog. Fish labelled with similar variants of the common name were considered correctly labelled. For example, *Acanthistius pictus* (common name: Cherlo) labelled as Mero Cherlo was considered correctly labelled (for additional examples see Table 2).

**Table 2:** Examples of common names of fish that were considered correctly labelled and mislabelled in markets and restaurants in Lima, Peru.

Chirichigno and Cornejo (2001)		Sold as…		FishBase
Scientific name	Common name Spanish Peru	Common names considered accurate	Common names considered mislabelled	Common name English
*Acanthistius pictus*	Cherlo, Choromelo, Calato, Chanchorro	Cherlo, Mero Cherlo		Brick Seabass
*Centropomidae*		Robalo	Lenguado	Snooks
*Cynoscion *sp.	Ayanque		Cachema, Charela, Cherela, Corvina	Whitefin Weakfish
*Oreochromis mossambicus*	Not included in the Catalog		Fortuno	Mozambique Tilapia
*Xiphias gladius*	Pez Espada, Albacora (in the South)	Diamante Albacora, Pez Espada	Tollo Diamante	Swordfish
*Isurus oxyrinchus*	Diamante, Mako, Tiburón Bonito	Diamante Albacora, Tollo Diamante	Tollo de Leche	Shortfin Mako
*Alopias pelagicus*	Not included in the Catalog	Tollo Zorro	Diamante Albacora, Tollo de Leche	Pelagic Thresher
*Alopias superciliosus*	Tiburón Zorro Ojón		Tollo	Bigeye Thresher
*Mustelus lunulatus*	Tollo	Tollo, Tollo de Leche		Sicklefin Smooth-hound
*Prionace glauca*	Tintorera, Azulejo, Verde Mar	Tiburón Azul, Tollo Azul	Cabrilla, Perico, Tollo, Tollo Bebé, Tollo Blanco, Tollo Cachito, Tollo de Leche, Tollo Diamante	Blue Shark

Sharks represent a special case when it comes to mislabelling. “Tiburón” (*shark* in Spanish) and “Tollo” are used almost interchangeably, with the latter being more commonly used. In Peru and Ecuador, Tollo is a term used for a variety of shark species in the families Carcharhinidae, Scyliorhinidae, Squalidae, and Triakidae^12,51^. Tollos are also commercialized as Tollo Bebé and Tollo de Leche^21,51^. Therefore, we considered Tollo, Tollo Bebé, and Tollo de Leche as the same common name. Because of this, we expected to find several species of sharks under the generic tollo label, and we do not consider most of those as being mislabelled. The only exception was if a species was commercialized as Tollo, Tollo de Leche, or Tollo Bebé but the species has a specific common name. For example, the common names for Shortfin Mako (*Isurus oxyrinchus*) are Diamante, Mako, and Tiburón Bonito, therefore, the generic label Tollo de Leche was not accepted as correct, but some more specific variants (e.g. Tollo Diamante) were considered correct (Supplementary Table S2).

### Mercury methods

Frozen muscle samples were dried at 60 °C for 48 h prior to total Hg analysis (MeHg + inorganic Hg). Total Hg was analysed using direct mercury analysis (Milestone DMA-80 Direct Hg Analyzer) which uses thermal decomposition, gold amalgamation, and atomic absorption spectroscopy^52^. Hg concentrations were converted from ng/g dry weight to ng/g wet weight by dividing dry weight by 4.7^53^. Quality assurance included reference (National Research Council of Canada Institute for National Measurement Standards) and duplicate samples. Reference samples (DORM-4 and PACS-3) were analysed every 10 samples, and the mean recovery percentage for DORM-4 was 93.9% (range, 87.9–99.81%; n = 21). Mean recovery percentage for PACS-3 was 96.6% (range 76.44–108.75%; n = 18). Duplicate samples were analysed every 20 samples, and the mean relative difference percentage was 9.23% ± 8.12% (range 0.04–28.22%; n = 20) (Supplementary Table S4). Total mercury (THg) was used as a proxy for methylmercury (MeHg) since previous studies have found that most of the THg in fish tissue is MeHg (i.e., > 95%^54^; 83.5 ± 19.7%^35^; 68.1–88.8%^34,36^). The assumption that > 90% of THg is MeHg applies to predatory species but does not necessarily apply to young fish and to fish from lower trophic levels where the percentage of MeHg is lower^35^. Thus, assuming that > 90% of THg is MeHg for lower trophic level fish (i.e., Flathead Grey Mullet) would overestimate the risk to consumers. Some raw (n = 2), marinated with lemon (e.g., ceviche; n = 5), and cooked (n = 9) samples from restaurants were also analysed for Hg. Previous research has found that different cooking methods have a dissimilar effect on the concentration of Hg on wet weight compared to concentrations in raw tissue. An increase in Hg concentration in cooked fish muscle has been attributed to the loss of moisture^55,56^. However, we included the cooked samples in the overall mercury analysis because while cooked samples may have lost some of the moisture, drying all samples prior to the Hg analysis resulted in a complete loss of moisture^55^.

### Mercury

Peru established a screening value of 1000 ng/g ww for “carnivorous” fish, whereas 500 ng/g ww was established for the rest of marine and freshwater species^15^. These values are the same as those assigned by the international food standards of the Codex Alimentarius^57^. In contrast, the US EPA has set its guidelines at 300 ng/g ww^20^. The US EPA recommended levels are lower than those recommended by Peru and FAO-WHO, in part, due to the inclusion of an uncertainty (or safety) factor to account for human-population variability and the lack of data on long-term effects of exposure^36^. We use this more conservative level to evaluate mercury content of seafood in this paper.

### By price

Prices from 48 species were used to determine if mislabelling and mercury concentrations varied by price range. Prices used correspond to wholesale prices (Peruvian Nuevos Soles [PEN], PEN/kg) in the wholesale fish market Villa Maria del Triunfo, in Lima^58,59^ (Supplementary Table S5). Since the price lists were limited, we extended the prices to species commercialized under the same common name. For example, only the price for Whiptail Stingray (*Dasyatis brevis*) was listed so we assigned the same price for the Chilean Eagle Ray (*Myliobatis chilensis*), since they are commonly commercialized simply as Ray (“Raya”). Based on price, the samples were grouped into four categories: (1) Below the 25th percentile (1–4.3 PEN); (2) Interquartile (4.6–12.3 PEN); (3) Above the 75th percentile (13.6–42.5 PEN); and (4) No price available.

### Comparisons with the two initiatives

To determine the efficacy of current campaigns for consuming sustainable seafood we compared the data from the samples collected (only those identified to species or genus) to the recommendations of the seafood guides bóVEDA and SDM. BóVEDA is a mobile phone application launched in 2014 that provides information about marine species including minimum landing size, seasonal harvest restrictions, and a list of ‘responsible restaurants’^9^. The bóVEDA app has a section called *Do not enter into temptation* that includes six species to avoid due to several factors (e.g., overfishing). The six species are Yellowfin Tuna, Corvina Drum, Striped Marlin, Peruvian Hake, Swordfish, and Hammerhead Shark (scientific names are not included in this section). BóVEDA does not include a best choice list; however, there is a section called *Size does matter* which includes a list of 15 invertebrates and 38 fish and the minimum size at which they should be consumed. We removed three species of fish from this section that were also included the ‘*Do not enter into temptation*’ list and considered these 35 species as ‘best choice’ from bóVEDA. SDM is a consumer seafood guide released in 2013 that gives minimum size limits for some of the most popular fish species and information about the best sustainable choices^8^. The SDM guide included 29 species of fish, divided into three categories: ‘best choice’ (15 species), ‘consume with caution’ (10 species), and ‘avoid’ (7 species) (Supplementary Table S6). We then merged the two lists for a final “best choice” list to use in our analyses. First, we removed five species from the bóVEDA best choice list because they were considered “avoid” or “consume with caution” in the SDM guide. We also added three species to this list because the Catalog^12^ lists them as having the same common names as the ones used in the seafood guides. Of the final list of 36 species, 12 were included in both lists, 18 were only listed in bóVEDA, three were only listed in SDM, and three are the extra ones we added due to the sharing of common names.

### Statistical methods

All mercury data were Log10 transformed before conducting t-tests (assuming unequal variances) and ANOVAs to meet test assumptions. Frequency data was analysed using Fisher’s exact tests and chi-square tests. All means are reported as untransformed ± (SE) standard error. Tests were conducted in Minitab 18 Software.

## Supplementary information

Supplementary Tables.
